# Anastomosing hemangioma of the kidney: a case report

**DOI:** 10.3389/fonc.2026.1767412

**Published:** 2026-06-19

**Authors:** Zhongyun Ning, Jinxia Liu, Shaolong Zhang, Chuanjian Chen, Ning Fan, Hui Ding

**Affiliations:** 1 Department of Urology, The Second Hospital of Lanzhou University, Lanzhou, China; 2 Department of Gynecology, The Second Hospital of Lanzhou University, Lanzhou, China

**Keywords:** anastomosing hemangioma (AH), benign, kidney, misdiagnosis, nephrectomy

## Abstract

Anastomosing hemangioma (AH) is a rare benign vascular neoplasm. It has been reported most commonly in the testes and genitourinary system [1-2], but it has also been described in the liver [3], gastrointestinal tract, ovary , and paraspinal region [4]. Although AH is well differentiated and has no malignant potential, it lacks specific imaging features and is therefore difficult to diagnose preoperatively. AH occurring in the liver is prone to be misdiagnosed as liver angiosarcoma [5]. Similarly, AH arising in the kidney is particularly difficult to distinguish from clear cell renal cell carcinoma, angiomyolipoma, and angiosarcoma, often leading to preoperative misdiagnosis. A 77-year-old woman was admitted to the Department of General Surgery with intermittent black stools for 20 days.After admission, she underwent an abdominal examination. Contrast-enhanced abdominal CT revealed a cystic lesion in the left renal sinus, approximately 2.0 cm × 2.0 cm in size. No significant enhancement was observed within the cyst on enhanced scanning, whereas nodular enhancement was visible in the cyst wall, raising the possibility of cystic renal carcinoma .laparoscopic radical nephrectomy was performed after exclusion of surgical contraindications. A diagnosis of anastomosing hemangioma of the kidney was made.

## Background

Anastomosing hemangioma (AH) is a rare benign vascular neoplasm. It has been reported most commonly in the testes and genitourinary system (1, 2), but it has also been described in the liver (3), gastrointestinal tract, ovary, and paraspinal region (4). Although AH is well differentiated and has no malignant potential, it lacks specific imaging features and is therefore difficult to diagnose preoperatively. AH occurring in the liver is prone to be misdiagnosed as liver angiosarcoma (5). Similarly, AH arising in the kidney is particularly difficult to distinguish from clear cell renal cell carcinoma, angiomyolipoma, and angiosarcoma, often leading to preoperative misdiagnosis.

## Case presentation

A 77-year-old woman was admitted to the Department of General Surgery with intermittent black stools for 20 days. After admission, she underwent an abdominal examination. Contrast-enhanced abdominal CT revealed a cystic lesion in the left renal sinus, approximately 2.0 cm × 2.0 cm in size. No significant enhancement was observed within the cyst on enhanced scanning, whereas nodular enhancement was visible in the cyst wall, raising the possibility of a cystic renal carcinoma (**Figures 1A, B**). The R.E.N.A.L. score was 9x. The patient also underwent an ultrasound gastroscopy. The results showed no abnormalities in the stomach and duodenal mucosa. There was a protruding lesion in the duodenum, which was considered a lipoma. The size of the lesion was 15.6 mm ×18.8 mm. After discussion with the general surgeon, the patient requested transfer to the Department of Urology for surgical management of the renal tumor.

**Figure 1:**
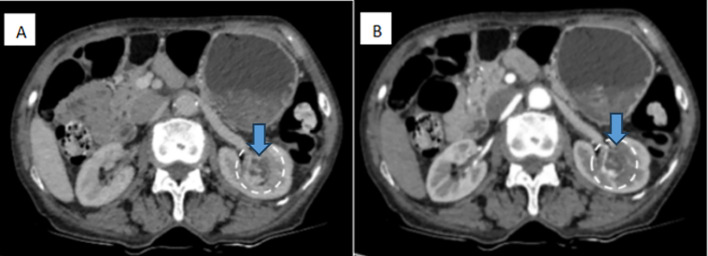
Relevant clinical data for the patient. **(A)** Renal CT showed that both kidneys were normal in size and shape. A multilocular cystic lesion was seen in the left renal sinus, approximately 2.0 cm × 2.0 cm in size. **(B)** No significant enhancement was observed within the cyst on enhanced scanning, but the cyst wall showed nodular enhancement. No enlarged retroperitoneal lymph nodes were identified. The dotted line and arrow indicate the lesion site.

After transfer to our department, her medical history was reviewed carefully. She had no urinary frequency, urgency, dysuria, or gross hematuria. Urinalysis indicated no microscopic hematuria. The tumor was a multilocular cystic mass located in the left renal sinus, measuring approximately 2 cm × 2 cm. After a multidisciplinary team (MDT) discussion at our hospital, the patient was initially diagnosed with cystic renal carcinoma. However, because the tumor was located in the renal sinus and was close to the renal artery, renal vein, and collecting system, partial nephrectomy was considered technically difficult. Therefore, a radical nephrectomy was planned, and informed consent was obtained from the patient and her family.

## Findings of the operation and pathology

After cardiopulmonary evaluation and assessment of bilateral glomerular filtration rates, laparoscopic radical nephrectomy was performed following confirmation that no surgical contraindications were present. The procedure was uneventful. No obvious perirenal adhesions or significantly enlarged hilar lymph nodes were observed.

During the operation, the kidney was completely mobilized, and the tumor was found to be entirely endophytic. Postoperative pathological examination showed that the tumor was composed of numerous proliferating small blood vessels, and the vascular endothelial cells showed no obvious atypia. The vessels formed a reticular anastomosing pattern, accompanied by hemorrhage and necrosis, and areas of transitional epithelial proliferation were observed. Based on the pathological changes and immunohistochemical findings, the lesion was differentiated from clear cell renal cell carcinoma, angiomyolipoma, and angiosarcoma. A diagnosis of anastomosing hemangioma of the kidney was made. The tumor did not invade the renal capsule, perirenal fat, or renal sinus fat. Immunohistochemical staining showed the following profile: CKp (−), EMA (−), CK7 (−),S100 (weakly +), CAIX (−), Vimentin (+), CD10 (−), HMB-45 (−), CD117 (−), TFE3 (−), S100 (−), Syn (−), CD56 (−), NSE (−), α-inhibin (−), endothelial CD34 (+), and CD31 (+), Pax-8 (−) (**Figure 2**). Approximately 5% of cells were positive for Ki-67. These findings supported the diagnosis.

**Figure 2:**
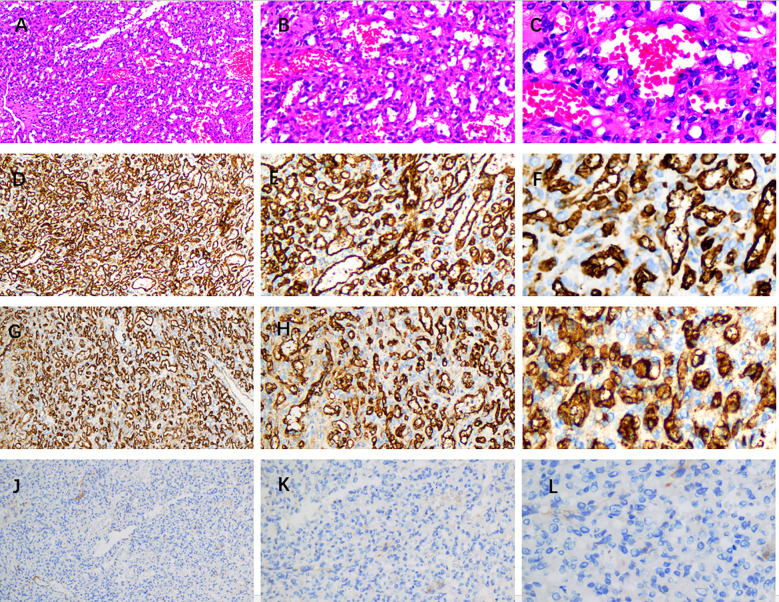
**(A-C)** H&E, ×100, ×200, ×400; **(D-F)** CD31, ×100, ×200, ×400; **(G-I)** CD34, ×100, ×200, ×400; and **(J-L)** PAX8, ×100, ×200, ×400. The tumor was composed of numerous proliferating small blood vessels, with no obvious atypia in the vascular endothelium, and the vascular channels formed an anastomosing network. The immunohistochemical indicators CD31 and CD34 showed strong positivity. The expression of PAX8 was negative.

## Discussion and conclusions

In summary, AH is a rare benign vascular lesion with a favorable prognosis. However, because of its clinical and radiological presentation, it can easily be misdiagnosed as clear cell renal cell carcinoma or other malignant tumors. Definitive diagnosis relies on careful pathological evaluation. In recent years, AH has also been reported in the ovary, adrenal gland, liver, gastrointestinal tract, mesentery/peritoneum, breast, bladder, soft tissue, and bone (6). In the 2020 WHO Classification of Soft Tissue Tumours, AH is recognized as a distinct benign vascular tumor (7). Most renal AHs occur in the renal parenchyma (70%), mainly in the medulla (50%). Less common sites include both the medulla and cortex (13%), the cortex alone (13%), the renal sinus (13%), perirenal fat (17%), and the renal vein (13%) (8). Sanger sequencing has shown that most cases harbor recurrent activating mutations in GNAQ, GNA11, and GNA14, indicating that AH is a true clonal vascular tumor (9–11). When the nature of the occupying lesion cannot be determined, these markers can be used as auxiliary diagnostic reference.

As an excellent mimic of renal tumors, AH usually presents with well-defined borders and no evidence of surrounding infiltration or tumor necrosis; however, imaging findings alone are insufficient to distinguish it from other renal tumors. In addition, in patients with renal AH who have good compliance, the cases reported by Sasaki et al. (12) and Zhang et al. (13) showed that renal AH typically grows slowly, suggesting that conservative management may be feasible. For patients in whom AH is suspected or whose lesions are difficult to diagnose preoperatively, biopsy may be considered (14).

## Data Availability

The raw data supporting the conclusions of this article will be made available by the authors, without undue reservation.
